# Maternal obstetric and socio-demographic determinants of low birth weight: a retrospective cross-sectional study in Ghana

**DOI:** 10.1186/s12978-019-0742-5

**Published:** 2019-05-29

**Authors:** Shamsudeen Mohammed, Irene Bonsing, Ibrahim Yakubu, Winifred Porsaa Wondong

**Affiliations:** 1 Department of Nursing, College of Nursing and Midwifery, P. O. Box 10 Nalerigu, Ghana; 2 Emergency Unit, Sunyani Municipal Hospital, Sunyani, Ghana; 3 Department of Nursing, Nursing and Midwifery Training College, Gushegu, Ghana

**Keywords:** Birth weight, Obstetric factors, Socio-demographic factors, Low birth weight, Determinants, Ghana

## Abstract

**Background:**

Birth weight is an important predictor of early neonatal mortality, morbidity, and long-term health outcomes. Annually, approximately 20 million babies are born globally with weights less than 2.5kg. In sub-Saharan Africa, the prevalence of LBW is around 13 to 15 percent. In Ghana, 10% of babies born in 2014 were with LBW. The aim of this study was to identify maternal socio-demographic and obstetric risk factors associated with the birth weight of newborns in the Sunyani Municipality of Ghana.

**Methods:**

This retrospective cross-sectional study analysed data from 931 birth records of all deliveries between January 1 and December 31, 2017, at the Sunyani Municipal Hospital in the Brong-Ahafo Region of Ghana. Univariate and multivariable logistic regression models were fitted to estimate the effect of maternal factors on low birth weight.

**Results:**

We found that the mean age of the participants and the mean gestational age at birth were 27.21(SD = 5.50) years and 37.95(SD = 1.85) weeks respectively. Nearly 10% of the infants born within the study period had birth weights below 2.5kg. The findings revealed that the odds of delivering LBW baby were significantly high (OR 1.77, 95%CI 1.14-2.76) among urban dwellers. However, mothers who attended or completed secondary or higher education were 63% (95% CI 0.20–0.78) less likely to give birth to a LBW baby when compared with uneducated mothers. We found that the odds of LBW significantly decreased with every one-week increase in gestational age (OR 0.67 95%CI 0.59-0.76) and significantly increased with increasing parity (OR 1.43 95%CI 1.21-1.70). Further, the likelihood of delivering LBW baby decreased with every additional ANC visit (OR 0.78 95%CI 0.67-0.90) and with every additional gram of haemoglobin (OR 0.78 95%CI 0.63-0.95).

**Conclusion:**

The evidence from this study suggests that maternal educational level, residence, haemoglobin level, parity, number of ANC visits, and gestational age are independent predictors of low birth weight. The current findings add substantially to the growing literature on the influence of maternal socio-demographic and obstetric factors on LBW in resource-constrained settings and provide empirical data for clinical and public health interventions aimed at reducing low birth weight and its associated complications.

## Plain English summary

At birth, most term newborns weigh between 2.5 kg and 4.0 kg. Those with an abnormally low or abnormally high birth weight require special attention and care to prevent neonatal death. According to the World Health Organization, the risk of neonatal death is much higher among neonates with low birth weight (LBW) than those with normal birth weight since LBW babies are more susceptible to birth asphyxia, trauma, hypothermia, hypoglycaemia, respiratory disorders, and infections [1]. Notwithstanding these immediate health consequences, LBW has long-term consequences in the form of growth inhibition, impairment of cognitive development, and increased incidence of chronic diseases such as type 2 diabetes, hypertension and cardiovascular diseases [2]. The 2014 Ghana Demographic and Health Survey reported that of those newborns weighed at birth, 10% had a LBW. We analysed data from birth records to identify the maternal factors associated with LBW in the Sunyani Municipality of Ghana. We found that maternal educational level, residence, haemoglobin level, parity, number of ANC visits, and gestational age are the socio-demographic and obstetric determinants of LBW in the municipality. The current findings add substantially to our understanding of the determinants of birth weight and provide empirical data for clinical and public health interventions aimed at reducing LBW and associated complications.

## Background

Birth weight is the first weight of a newborn after delivery. It is an important predictor of early neonatal mortality, morbidity, and long-term health outcomes [2]. At birth, most term newborns weigh between 2.5 kg and 4.0 kg. Those with an abnormally low or abnormally high birth weight require special attention and care to prevent neonatal death [3]. Newborns with a birth weight of less than 2.5 kg are considered to be of LBW (LBW), irrespective of the gestational age. Annually, approximately 20 million babies are born globally with weights less than 2.5 kg. In sub-Saharan Africa, the prevalence of LBW is around 13 to 15% [2]. The 2014 Ghana Demographic and Health Survey reported that of those newborns weighed at birth, 10% had a LBW [4]. Studies have shown that LBW is the result of either a short gestation period (less than 37 completed weeks of gestation) or intrauterine growth restriction. These two major factors of LBW have varied aetiology and risks of mortality and morbidity [5]. In 2010, approximately 32 million babies were born with LBW in low and middle-income countries of which 2.8 million of them were preterm. Furthermore, Of the 18 million LBW babies born each year, approximately 59% are due to intrauterine growth restriction in term infants and 41% are attributable to prematurity [5]. Globally, a huge burden of foetal growth restriction exists and babies born at term with low birth weight are linked to intrauterine growth restriction. For instance, in 1998 and in 2010, about 13.7 million and 10.6 million infants were born at term with low birth weight in low and middle-income countries respectively and these LBWs were attributed to intrauterine growth restrictions [5].

Evidence have demonstrated that LBW has significant consequences for the health and survival of neonates and is an underlying factor in neonatal deaths [6]. According to the World Health Organization, the risk of neonatal death is much higher among neonates with LBW than those with normal birth weight since LBW babies are more susceptible to birth asphyxia, trauma, hypothermia, hypoglycaemia, respiratory disorders, and infections [1]. Notwithstanding these immediate health concerns, LBW has long-term consequences in the form of growth inhibition, impairment of cognitive development, and increased incidence of chronic diseases such as type 2 diabetes, hypertension and cardiovascular diseases [2, 7]. Additionally, findings of the 2014 Ghana Demographic and Health Survey suggests that children whose birth weight is less than 2.5 kg have a higher than average risk of early childhood death [4].

In many low-income countries, LBW is associated with lower socioeconomic status, female sex, increasing parity, young maternal age, low level of maternal education, low caloric intake, malaria, and general morbidity [8–10]. Furthermore, studies have demonstrated a link between ABO blood groups and birth weight [11, 12]. For instance, in Turkey, Beyazit et al. reported that pregnant women with type B blood group had significantly lower birth weight babies compared with women with other ABO blood groups [11]. Further, a case control study in Nepal found that maternal blood group AB had some protective effect against delivering a LBW baby [13]. Other potentially important factors in determining newborn weight are maternal haemodynamics, antenatal care visits, folic acid intake, and quality of antenatal care [8–10]. Several studies in low and middle-income countries have shown empirical support for the link that exists between maternal factors and birth weight [14–17]. However, few studies have been conducted on maternal determinants of LBW in the Sunyani Municipality of Ghana. To modify the risk factors of LBW and prevent the life-long complications associated with it, there is the need to understand the determinants of LBW in the municipality. This study was undertaken to identify maternal socio-demographic and obstetric risk factors associated with the LBW of newborns in the Sunyani Municipality of Ghana and to provide empirical data for clinical and public health interventions aimed at preventing LBW and associated complications.

## Methodology

### Study setting and study design

A retrospective cross-sectional study was conducted in Sunyani Municipal Hospital in the Brong-Ahafo Region of Ghana. The hospital was established in 1927 and upgraded to the status of a municipal hospital in 2004. It is located in the Sunyani Municipal Assembly, one of the 27 districts in the Brong Ahafo Region. The Sunyani Municipal Assembly has a total land area of 506.7 Km^2^. Six hospitals, 12 clinics, seven chips compound, three maternity homes, and three health centres provide health care services to the municipal population of 123,224 (61,610 males and 61,614 females) [18].

The Sunyani Municipal Hospital provides 24 h uninterrupted healthcare services, including specialised services for both insured and uninsured patients from diverse ethnic and socioeconomic backgrounds. It serves as one of the referral centres for the primary level health care facilities in rural and urban communities of the municipality. In 2017, 63,043 outpatients reported at the OPD of the hospital of which 6821 were admitted. Antenatal care attendance in the hospital declined from 10,813 in 2016 to 9966 in 2017 with a similar decline in the number of women who made four or more visits (1889 in 2016 to 1249 in 2017). Professional nurses and midwives in the maternity unit of the hospital record pregnancy history of mother’s, newborn characteristics, delivery information, and socio-demographic characteristics of all mothers in a birth register. We examined birth records of all mothers who delivered live babies in the Sunyani Municipal Hospital between January 1 and December 31, 2017, to determine the influence of maternal socio-demographic and obstetric factors on the birth weight of newborns.

### Data extraction and sample size

Data for the study were extracted from birth registers containing information about maternal and newborn characteristics using a structured data-capturing sheet. The data-capturing sheet was pretested on birth records for March 2018 and appropriate amendments made. Three Registered Nurses (RN) extracted the data from the registers. They were trained on how to extract data from the records and orientated on the eligibility criteria of the study. The second author (IB) supervised the data collectors on daily basis to ensure completeness and consistency of data extraction and allowed frequent scheduled breaks to prevent errors. Permission to use the birth records was granted by the medical superintendent of the hospital. Newborn deliveries recorded in the maternity unit of the hospital between January 1 and December 31, 2017, were 1756. All the newborn deliveries within this period were considered in this study. However, we analysed data from 931 records after excluding records with multiple births, stillbirths, babies with congenital abnormalities, and entries with missing birth weight information. Stillbirths were excluded because the birth weights of the few cases of stillbirths that were recorded within the study period were missing. We excluded records of multiple births and congenital abnormalities because they have a different aetiology and risk profile for LBW [19].

### Study variables and categorisation

#### Outcome variable

The outcome variable in this study was birth weight. Newborn records with birth weight less than 2.5 kg were considered LBW while those with a birth weight of 2.5 kg or more were considered as normal birth weight, irrespective of gestational age. Based on these criteria the outcome variable was extracted and coded as a dichotomous variable: 0 “LBW (< 2.5 kg)” and 1 “normal birth weight (≥2.5kg)”.

#### Explanatory variables

Explanatory variables of this study were maternal socio-demographic characteristics and obstetric factors that may influence the birth weight of newborns, given the results of similar previous studies [9, 20–24]. Socio-demographic characteristics that were captured from the records were maternal age in completed years, mother’s education level, occupation, and residence. Maternal age at birth was categorised into three groups: < 20, 20–30, and > 30. Education level refers to the highest level of schooling attended by the mother whether completed or not. Education level was classified as uneducated, primary/Junior High School (JHS), and secondary or higher education. Occupation of mothers was classified as employed (government/private), self-employed, unemployed and student. Residence of mothers was coded into two categories: 0 ‘rural’ 1 ‘urban’.

Maternal factors that were extracted were gravida, parity, number of ANC visits, gestational age, intake of Intermittent Preventive Treatment in pregnancy with sulfadoxine-pyrimethamine (IPTp-SP), haemoglobin level (Hb), and blood group. Gravida was categorised as primigravida, gravida 2–4 and gravida 5 or more. Parity did not include current birth and was categorised as first-time mothers, 1–3 previous deliveries, and ≥ 4 previous deliveries. The total number of antenatal care visits for the pregnancy was classified as ≤4 visits and > 4 visits. Gestational age was calculated based on the last menstrual period (LMP) or antenatal ultrasound assessment if the LMP was not known. According to WHO, half of babies born at or below 32 weeks in low-income settings die [25]. In this study, gestational age was classified as 26–36 weeks and 37–42 weeks. IPTp-SP was categorised as none (for mothers who did not take a dose of IPT-SP), 1–3 doses, and ≥ 4 doses. Haemoglobin level of mothers was binary coded based on WHO recommendation [26] as no-anaemia (≥11 g/dl) and anaemia (< 11 g/dl). Blood group was categorised based on the ABO system as A, B, AB, and O.

### Statistical analysis and data management

Data capture sheets were checked for completeness and accuracy. Data were then coded, entered into Microsoft Excel spreadsheet, and imported into STATA Version 14.0 (College Station, Texas, USA) for cleaning and analysis. Frequencies, percentages, means, or standard deviations were used to describe maternal socio-demographic and obstetric characteristics. Univariate logistic regression models were fitted to estimate the effect of maternal socio-demographic characteristics and maternal obstetric factors (independent variables) on LBW (dependent variable). Parameters that were significant (< 0.05) at the univariate level were included in the final multivariable logistic regression models to determine the independent predictors of LBW. Variables were retained in the final model if they were still significant (< 0.05) after adjustment. Statistical significance for all the statistical test was considered at < 0.05.

## Results

### Maternal socio-demographic characteristics

Table 1 presents the socio-demographic characteristics of mothers who delivered at the Sunyani Municipal Hospital between January 1 and December 31, 2017. Maternal mean age at delivery was 27.21(SD = 5.50) years. Six hundred and eight (66.4%) mothers were between ages 20 to 30 years. Teenage mothers were only seventy-eight (8.4%). Basic education (Primary/Junior High School) was the highest level of education attended or completed by most of the mothers (48.4%) and mothers with no formal education were one hundred and seven (11.5%). More than half (56.9%) of the mothers were self-employed. Mothers who were employed in the formal sector were one hundred and sixty-one (17.3%) while unemployed mothers were one hundred and forty-seven (15.8%). Most (58.8%) of the women were from rural communities within the study area.

**Table 1:** Socio-demographic characteristics of women delivering at Sunyani Municipal hospital between January 1 and December 31, 2017 (*N* = 931)

Characteristics	Number	Percent
Age of mother		
< 20	78	8.38
20–30	618	66.38
> 30	235	25.24
Mean (SD)	27.21 (5.50)	
Education		
No formal education	107	11.49
Basic education	451	48.44
Secondary or higher education	373	40.06
Occupation		
Employed (Government and private)	161	17.29
Self-employed	530	56.93
Unemployed	147	15.79
Student	93	9.99
Residence		
Rural	547	58.75
Urban	384	41.25

### Maternal obstetric characteristics

As shown in Table 2, the mean gestational age at birth was 37.95(SD = 1.85) weeks. Most of the women (*n* = 797; 85.6%) gave birth between 37 and 42 weeks gestation. Preterm birth was prevalent in one hundred and thirty-four (14.4%) mothers. The majority of the women (75.4%) were para 1–3; 15.4% (*n* = 143) were primiparous. Nearly three-quarters (73.4%) of the women had had 2 to 4 pregnancies and 84.9% of the women had more than four ANC visits during pregnancy. Most (71.9%) of the mothers received 1 to 3 doses of Intermittent Preventive Treatment with sulfadoxine-pyrimethamine (IPTp-SP) during pregnancy to prevent malaria. Less than half (40.1%) of the women were anaemic and blood group AB (41.6%) was the most common blood group among the mothers. The mean birth weight was 2.89(SD = 0.43) kg. The majority of the children born within the study period had normal birth weight (*n* = 842, 90.4%). Only 89(9.6%) children born within the study period had LBW.

**Table 2:** Obstetric characteristics of women delivering at Sunyani Municipal hospital between January 1 and December 31, 2017 (*N* = 931)

Characteristics	Number	Percent
Gestational age (weeks)		
26–36 weeks	134	14.39
37–42 weeks	797	85.61
Mean (SD)	37.95 (1.85)	
Parity		
First-time mother	143	15.36
1–3 previous deliveries	702	75.40
≥ 4 previous deliveries	86	9.24
Gravida		
Primigravida	144	15.47
Gravida 2–4	683	73.36
Gravida 5 or more	104	11.17
Number ANC visits		
≤ 4 visits	140	15.04
> 4 visits	791	84.96
Number of SP doses		
None	22	2.36
1–3 doses	669	71.86
≥ 4 doses	240	25.78
Haemoglobin		
No-anaemia	558	59.94
Anaemia	373	40.06
Blood group		
A	253	27.18
B	247	26.53
AB	387	41.57
O	44	4.73
Birth weight of child		
≥ 2.5 kg (Normal birth weight)	842	90.44
< 2.5 kg (LBW)	89	9.56
Mean (SD)	2.89 (0.43)	

### Socio-demographic determinants of LBW

The association between birth weight and age of mother, education, occupation, and residence of mothers is shown in Table 3. The residence status of mothers and their educational level were the only variables significant in the unadjusted model. After adjusting for the residence of mothers, secondary or higher education remained a significant (*p* = 0.008) predictor of birth weight and mothers who attained this level of education were 63% (95% CI 0.20–0.78) less likely to have LBW infants. As shown in Table 3, the odds of delivering a LBW baby in the adjusted (OR 1.77, 95% CI 1.14–2.76) logistic regression model was significantly high among urban dwellers.

**Table 3:** Association between socio-demographic characteristics of women delivering at Sunyani Municipal hospital between January 1 and December 31, 2017 and LBW (*N* = 931)

Characteristics	Unadjusted model		Adjusted model^a^	
	OR (95% CI)	*P* value	OR (95% CI)	*P* value
Age of mother (years)				
< 20	1.39 (0.68, 2.85)	0.364		
20–30	Ref			
> 30	0.88 (0.51, 1.49)	0.641		
Education				
No formal education	Ref		Ref	
Basic education	0.70 (0.38, 1.30)	0.267	0.73 (0.39, 1.34)	0.315
Secondary and higher education	0.37 (0.19, 0.73)	0.005	0.39 (0.20, 0.78)	0.008
Occupation				
Employed (Government and private)	Ref			
Self-employed	1.10 (0.58, 2.10)	0.754		
Unemployed	1.39 (0.64, 2.99)	0.401		
Student	1.85 (0.81, 4.18)	0.139		
Residence				
Rural	Ref		Ref	
Urban	1.85 (1.19, 2.87)	0.006	1.77 (1.14, 2.76)	0.011

^a^Predictors adjusted for in the model were education and residence

### Obstetric determinants of LBW

Table 4 presents logistic regression models of the association between birth weight and maternal obstetric factors. As shown in the unadjusted model, the odds of LBW significantly decreased with every one-week increase in gestational age (OR 0.65, 95% CI 0.57–0.73) and significantly increase with increasing parity (OR 1.42, 95% CI 1.21–1.66). The odds of LBW decreased with every additional ANC visit (OR 0.71, 95% CI 0.62–0.80), IPTp-SP dose (OR 0.75, 95% CI 0.61–0.93) and a gram of haemoglobin (OR 0.73, 95% CI 0.61–0.89). After adjusting for the effect of other significant covariates, gestational age (OR 0.67 95% CI 0.59–0.76), parity (OR 1.43 95% CI 1.21–170), number of ANC visits (OR 0.78 95% CI 0.67–0.90), and maternal haemoglobin level (OR 0.78 95% CI 0.63–0.95) remained significant predictors of LBW.

**Table 4:** Association between obstetric characteristics of women delivering at Sunyani Municipal hospital between January 1 and December 31, 2017 and LBW (*N* = 931)

Characteristics	Unadjusted model		Adjusted model^a^	
	OR (95% CI)	*P* value	OR (95% CI)	*P* value
Gestational age (weeks)	0.65 (0.57, 0.73)	< 0.001	0.67 (0.59, 0.76)	< 0.001
Parity	1.42 (1.21, 1.66)	< 0.001	1.43 (1.21, 1.70)	< 0.001
Gravida	0.86 (0.72, 1.02)	0.092		
Number ANC visits	0.71 (0.62, 0.80)	< 0.001	0.78 (0.67, 0.90)	0.001
Number of IPTp-SP doses	0.75 (0.61, 0.93)	0.011		
Haemoglobin	0.73 (0.61, 0.89)	0.002	0.78 (0.63, 0.95)	0.016
Blood group				
A	Ref			
B	0.71 (0.39, 1.29)	0.272		
AB	0.74 (0.44, 1.25)	0.268		
O	0.99 (0.36, 2.71)	0.985		

^a^Predictors adjusted for in the model were gestational age, parity, number of ANC visits, Number of IPTp-SP doses, and haemoglobin

## Discussions

We found that nearly 10% of the infants born within the study period had birth weights below 2.5kg (LBW). Further analysis revealed that the odds LBW were higher among babies born to teenage mothers (age less than 20 years) and low among babies of mothers older than 30 years albeit not significant, which agrees well with several other studies [23, 27–33] including findings of the 2010 Ghana Demographic and Health Survey [4]. This may be due to poor socioeconomic status, maternal malnutrition, and inadequate antenatal care of teenage mothers as these factors have been reported to influence birth weight of babies born to teenage mothers in low-and-middle-income countries [34–36]. Additionally, biological immaturity and other behavioural factors may have aggravated the increased risk of LBW among teenage mothers. A cohort study in five low-and-middle-income countries found that babies born to teenage mothers have a double risk of LBW [27], which further supports the finding of the current study. Despite the strong evidence supporting the effect of young maternal age on LBW, there are some other studies that did not find any relationship between maternal age and LBW [37, 38].

Mothers who attended or completed basic education and secondary or higher education were less likely to give birth to a low weight baby when compared with those with no formal schooling. Indeed, after adjusting for the residence of mothers, secondary or higher education remained a significant predictor of birth weight and mothers who attained this level of education were 63% less likely to have LBW infants. This finding is congruent with several studies which link LBW to the educational level of mothers [10, 31, 39]. A possible explanation for this might be that increased years of maternal education brings about improvement in ANC attendance, nutritional status, health-seeking behaviour and enhanced maternal experiences relative to pregnancy and childcare. Furthermore, increased years of maternal education may cause delay in sexual initiation or increase uptake of contraception to prevent pregnancy [40, 41]. This inadvertently could increase maternal age at first birth and decrease the likelihood of LBW associated with teenage pregnancy.

In this study, the likelihood of delivering a LBW baby was significantly high among urban dwellers, which is consistent with studies in Ethiopia [42] and Bangladesh [24]. It seems possible that these results may be due to urban women attitude toward antenatal care, interpregnancy interval, and iron and vitamin supplementation during pregnancy [24]. For instance, in Zambia, Banda et al. found that urban women did not believe that early commencement of ANC offers any benefits, they had inadequate knowledge about ANC, and negative cultural beliefs about early ANC attendance [43]. Given the relevance of early ANC attendance to maternal and neonatal health, these negative attitudes may account for the increased odds of LBW among urban women [44]. However, unlike this study, an earlier study in Ghana reported an increased risk of LBW among women from rural settings [9].

We found that with every one-week increase in gestational age, the odds of LBW significantly decreased. As observed in previous studies, pre-term babies are usually underweight, therefore, once delivery occurs at term the likelihood of a baby weighing above 2.5 kg is high [10]. Our findings further suggest that the odds of LBW significantly increase with increasing parity, which is in line with the findings of several authors [9, 15, 27]. Up-to-date there is no clear mechanism on how parity influences LBW [45, 46]. However, studies have shown that the increased occurrence of preeclampsia and the younger age of nulliparous women, the incidence of chronic anaemia, diabetes mellitus, and hypertension in multipara women, and the higher incidence of placenta previa, abruption, abnormal presentation, and haemorrhagic in grand multiparous women may reduce foetal growth and duration of pregnancy, which may predispose to LBW [45, 46].

Another significant finding from this study was that the probability of a woman delivering a LBW baby decreased with every additional ANC visit, IPTp-SP dose and a gram of haemoglobin. The significance of ANC in maternal and child health cannot be overemphasised. There is strong evidence to suggest that quality ANC during pregnancy is important for the health of the expectant mother and the developing foetus. This is ensured through essential interventions such as the identification and management of obstetric complications including preeclampsia, tetanus toxoid immunisation, intermittent preventive treatment for malaria during pregnancy (IPTp), and identification and management of infections including HIV, syphilis and other sexually transmitted infections (STIs) at the ANC [47]. However, poor and late ANC attendance is usually associated with increased odds of LBW [8]. A case-control study collaborated by WHO in Pakistan revealed that delivering a LBW baby decreased with an increase in maternal haemoglobin and that the odds were greater among mothers not using iron supplements during pregnancy [22]. Therefore, mothers of LBW babies had lower haemoglobin levels before delivery. This study agrees with our finding that an increase in haemoglobin reduces the likelihood of LBW.

The most important limitation of the current study lies in the fact that the data used for the analyses were primarily collected for routine healthcare services and not for research purposes or for a specific intervention. Errors may have occurred during the documentation of the records. Notwithstanding this limitation, routine collection of health data may allow for the monitoring and evaluation of public health interventions. Finally, the study analysed data from one hospital and findings may not be generalizable to mothers who attended other hospitals and those who delivered at home.

## Conclusion

The evidence from this study suggests that maternal educational level, residence, haemoglobin level, parity, number of ANC visits, and gestational age are independent predictors of LBW. These findings contribute to the growing literature on the influence of maternal socio-demographic and obstetric factors on LBW in resource-constrained settings. This could guide the development of clinical and public health interventions aimed at reducing LBW and its associated complications.

## Data Availability

The datasets used and/or analysed during the current study are available from the corresponding author on reasonable request.

## Abbreviations

ANC: Antenatal Care
IPTO-SP: Intermittent Preventive Treatment in pregnancy with sulfadoxine-pyrimethamine
JHS: Junior High School
LBW: Low Birth Weight
WHO: World Health Organisation
